# Effectiveness of a Mobile Health and Self-Management App for High-Risk Patients With Chronic Obstructive Pulmonary Disease in Daily Clinical Practice: Mixed Methods Evaluation Study

**DOI:** 10.2196/21977

**Published:** 2021-02-04

**Authors:** Laura Kooij, Petra J E Vos, Antoon Dijkstra, Wim H van Harten

**Affiliations:** 1 Rijnstate Hospital Arnhem Netherlands; 2 Division of Psychosocial Research and Epidemiology The Netherlands Cancer Institute Amsterdam Netherlands; 3 Department of Health Technology and Services Research University of Twente Enschede Netherlands

**Keywords:** chronic obstructive pulmonary disease, mHealth, self-management, mobile app, mobile phone

## Abstract

**Background:**

Mobile health and self-management interventions may positively affect behavioral change and reduce hospital admissions for patients with chronic obstructive pulmonary disease (COPD). However, not all patients qualify for these interventions, and systematic, comprehensive information on implementation- and compliance-related aspects of mobile self-management apps is lacking. Due to the tendency to target digital services to patients in stable phases of disease, it is especially relevant to focus on the use of these services in broad clinical practice for patients recently discharged from hospital.

**Objective:**

This study aims to evaluate the effects of a mobile health and self-management app in clinical practice for recently discharged patients with COPD on use of the app, self-management, expectations, and experiences (technology acceptance); patients’ and nurses’ satisfaction; and hospital readmissions.

**Methods:**

A prototype of the app was pilot tested with 6 patients with COPD. The COPD app consisted of an 8-week program including the Lung Attack Action Plan, education, medication overview, video consultation, and questionnaires (monitored by nurses). In the feasibility study, adult patients with physician-diagnosed COPD, access to a mobile device, and proficiency of the Dutch language were included from a large teaching hospital during hospital admission. Self-management (Partners in Health Scale), technology acceptance (Unified Theory Acceptance and Use of Technology model), and satisfaction were assessed using questionnaires at baseline, after 8 weeks, and 20 weeks. Use was assessed with log data, and readmission rates were extracted from the electronic medical record.

**Results:**

A total of 39 patients were included; 76.4% (133/174) of patients had to be excluded from participation, and 48.9% of those patients (65/133) were excluded because of lack of digital skills, access to a mobile device, or access to the internet. The COPD app was opened most often in the first week (median 6.0; IQR 3.5-10.0), but its use decreased over time. The self-management element knowledge and coping increased significantly over time (*P*=.04). The COPD app was rated on a scale of 1-10, with an average score by patients of 7.7 (SD 1.7) and by nurses of 6.3 (SD 1.2). Preliminary evidence about the readmission rate showed that 13% (5/39) of patients were readmitted within 30 days; 31% (12/39) of patients were readmitted within 20 weeks, compared with 14.1% (48/340) and 21.8% (74/340) in a preresearch cohort, respectively.

**Conclusions:**

The use of a mobile self-management app after hospital discharge seems to be feasible only for a small number of patients with COPD. Patients were satisfied with the service; however, use decreased over time, and only knowledge and coping changed significantly over time. Therefore, future research on digital self-management interventions in clinical practice should focus on including more difficult subgroups of target populations, a multidisciplinary approach, technology-related aspects (such as acceptability), and fine-tuning its adoption in clinical pathways.

**Trial Registration:**

Clinicaltrials.gov NCT04540562; https://clinicaltrials.gov/ct2/show/NCT04540562.

## Introduction

### Background

Chronic obstructive pulmonary disease (COPD) affects over 250 million people worldwide [1] and almost 600,000 people in the Netherlands [2]. In 2020, it is expected to be the third leading cause of death worldwide [3]. COPD is a common disease characterized by persistent respiratory symptoms and airflow limitation due to airway and/or alveolar abnormalities [3]. The most common symptoms are dyspnea, chronic coughing, and sputum production [3-5]. An acute worsening of the symptoms is called an exacerbation [4,6]. Exacerbations lead to additional care [5] and often lead to hospital admission [7], with considerable costs involved [8].

Self-management interventions are also recognized to be important in reducing exacerbations [9] and hospital admissions [10,11], improving quality of life [9-11], and improving patients’ control over their health [9]. Self-management skills can be beneficial for patients with COPD to manage their disease on a daily basis [12], for example, for medication use, breathing techniques, physical activity, and symptom recognition [13]. Effing et al [12] defined these interventions for patients with COPD as structured, personalized, and often multi-component, with goals of motivating, engaging, and supporting patients to positively adapt their health behaviors. Relevant features for self-management interventions include smoking cessation, recognition and treatment of exacerbation, increasing physical activity, nutrition advice, and management of dyspnea [14].

Mobile apps are increasingly being used to provide patients with health and self-management interventions, for example, for remote monitoring of patients’ health status [15-17], self-report of symptoms or health status [16-18], education [16,19], and digital support or feedback [15,17,18]. This is often combined with feedback from a health care professional or automated via the app [17-19]. Multiple reviews have analyzed the effectiveness of self-management interventions supported by mobile apps for patients with COPD on hospital admissions [15,18], exacerbations [15,16], length of hospital stay [18], behavioral outcomes [15,19], health-related outcomes [15,19], and quality of life [15]. The use of smartphones can be feasible in providing patients with self-management interventions [20,21] and to improve behavioral change [21]. A recent review reported the effects of smartphone interventions on exacerbations and showed that these interventions may decrease exacerbations, compared with usual care [16]. However, the findings remain inconsistent [17] due to heterogeneity among interventions [9,16,17,19,22], target populations [9,22,23], outcomes [9,22,23], and small sample sizes [16]. Further research and analysis on relevant apps for apps to support patients with COPD is necessary [24], as evidence is limited [15].

Until now, much attention has been given to the effects on clinical health outcomes [11,25-27] and hospital services [11,28,29]. Self-management behavior is also found to be important in reducing hospital admissions [30]. Factors affecting use in daily clinical practice, such as patients’ satisfaction [31], technology acceptance [32,33], and health care professionals’ satisfaction [34], were examined to a lesser extent. It also remains unclear which patients benefit most from these digital interventions [35,36]. It is suggested that it may be beneficial for patients experiencing frequent exacerbations [37]; nevertheless, stable patients with COPD are often the target population [38]. Patients experiencing a hospital admission due to an exacerbation may require a different approach, as they often experience feelings of distress during this time [39]. Additional evidence on this specific subpopulation is still needed [36], especially in combination with mobile health (mHealth) solutions [16]. Health care professionals’ involvement is also essential for a successful self-management intervention in clinical practice [13].

Self-management interventions, which are increasingly supported by mobile apps in recent years, may improve disease management in patients with COPD and may decrease hospital admissions. However, not all patients qualify because of reasons such as socioeconomic status, internet access, and skills. Systematic, comprehensive information on implementation- and compliance-related aspects of mobile self-management apps is lacking. Additional evidence about the effectiveness of mobile self-management apps is needed, especially regarding factors affecting the use in clinical practice for high-use patients, such as those recently hospitalized due to an exacerbation.

### Objectives

The objective of this study is to evaluate the effects of a mobile health and self-management app (*COPD app*) in clinical practice for patients with COPD, after discharge from the hospital, on app use, self-management, expectations and experiences (technology acceptance), patients’ and nurses’ satisfaction, and hospital readmissions.

## Methods

### COPD App

The COPD app consisted of an 8-week health and self-management intervention, including the Lung Attack Action Plan, personalized medication overview, information about COPD, nutrition, physical activity, advantages of smoking cessation, weekly questionnaires monitored by nurses, and video consultation.

### Pilot Testing

Pilot testing was used to receive feedback on a prototype of the COPD app. A total of 6 patients, admitted to a large teaching hospital (Rijnstate, Arnhem) for a COPD exacerbation, were provided with a tablet and access to the app. Patients received assignments such as *Can you find and use the Lung Attack Action Plan*, *Can you find and open the questionnaire*, and *Can you find and read the information about nutrition*. We also asked their opinion about the information (eg, if they missed information elements), frequency of notifications they would prefer, the readability, the frequency of new information, and their sociodemographic characteristics. Before starting the feasibility study, results from the pilot testing were used to improve the COPD app.

### Feasibility Study—Recruitment and Eligibility Criteria

Patients were recruited from a large teaching hospital (Rijnstate, Arnhem). To be eligible, patients must be older than 18 years, diagnosed for COPD by a physician, admitted to the hospital for a COPD exacerbation (generally considered high-risk patients), have access to a smartphone or tablet, have a working internet connection, being able to use a smartphone or tablet, and be proficiency in Dutch language. Patients with cancer or (severe) cognitive or psychiatric conditions were excluded. At least one hospitalization for COPD exacerbation in the year preceding this study was also a criterion for accrual, but it only applied during the first month (of the inclusion period) because the number of eligible patients was too low.

### Study Process

Patients were informed about the study by a pulmonary nurse and the researcher during hospital admission. Patients received the study information letter and were asked to sign the informed form. They also received support to download apps. The *Patient Journey App* software (PJA version 4.0) [40] was used for the COPD app and *Facetalk* [41] for video consultation. The apps could be downloaded for free from the Google Play Store and the Apple App Store [41-43].

### Intervention

The COPD app provided patients with an 8-week self-management program. The app had 3 views: timeline, information page, and contact page (see Multimedia Appendix 1). The start date was the date of discharge of each patient. The timeline was classified in 8 weeks, and each week included the Lung Attack Action Plan, personalized (daily and extra) medication overview, information and education, and questionnaires. The first week also included a video of a pulmonologist explaining the purpose of the app and additional information about the functionalities of the COPD app. After 8 weeks (until 20 weeks), patients remained accessible to the information in the app, but the questionnaires, medication overview, video consultation, and Lung Attack Action Plan (including contact request) were no longer accessible.

#### Timeline

The timeline consisted, in all weeks, of 5 elements: (1) *Lung Attack Action Plan*, (2) *Medication Overview*, (3) *Information and Education*, (4) *Questionnaires*, and (5) *Consultations*, in week 4 and 8 (see Multimedia Appendices 1 and 2).

#### Lung Attack Action Plan

The Lung Attack Action Plan was provided by the Lung Foundation (*Longfonds*) [44] and was digitalized in the COPD app. This action plan could help patients to recognize changes in their symptoms and guide them how to act upon these changes. The action plan consisted of different categories and colors: *I am doing well today* (green), *I feel worse* (yellow), *No improvement after 2 days* (orange), and *The situation is threatening* (red). All levels included advice about symptoms (eg, dyspnea, production of sputum, and coughing), medication, physical activity, and nutrition. Patients could access and use the Lung Attack Action Plan at any time using the COPD app. It was also possible to request contact with a pulmonary nurse after using the Lung Attack Action Plan. The nurse received a notification email and would contact patients within 2 working days.

#### Medication Overview

Patients had access to an overview of their personal daily and extra medication.

#### Information and Education

A total of 5 information categories were included in the timeline: the COPD app, the condition COPD, physical activity, nutrition, and advantages of smoking cessation. For each topic, a general page was accessible, including more specific topics. Patients were provided with information, in text and video, about the COPD app (eg, information about the different functionalities), COPD condition (eg, recognizing an exacerbation and accepting your lung condition), nutrition (eg, advice about protein-rich food), physical activity (eg, videos with exercises from a physiotherapist), and smoking cessation (eg, advantages of smoking cessation after 20 min and 1 month).

#### Questionnaires and Monitoring

Patients were asked to fill out the weekly Clinical COPD Questionnaire (CCQ) and the Hospital Anxiety and Depression Scale (HADS) at weeks 1 and 8, using the app or via email. The results were monitored by nurses. The HADS was used to measure anxiety and depression The HADS is a 14-item screening list that consists of two 7-item subscales. The items are rated on a 4 point Likert scale (range 0-3) [45,46]. The CCQ is a self-administered questionnaire used to assess patients’ clinical control. The CCQ is a 10-item scale with 3 domains: functional state, symptoms, and mental state, rated on a 7-point scale (0: no limitation to 6: totally limited). The CCQ score was calculated as the mean of the sum of all items [47]. The first CCQ was completed during hospital admission and repeated weekly. The nurses checked the scores weekly, and if a score was >2 and increased since the previous week, they contacted the patient.

#### Consultations

A video consultation was planned after 4 weeks with a pulmonary nurse, and a face-to-face consultation was planned after 8 weeks with a nurse practitioner or a pulmonologist. Patients could also request additional video consultations and telephonic consultations using the COPD app.

#### Information Page

The information page contained an overview of the information elements: Lung Attack Action Plan, the COPD app, condition COPD, nutrition, physical activity, smoking cessation, and information about video consultation. The information elements were presented in a list format, with a search function. See Multimedia Appendices 1 and 3.

#### Contact Page

The contact page presented 2 elements for patients: (1) the Lung Attack Action Plan and the option to request contact with a pulmonary nurse or (2) directly request telephonic contact with a nurse. Nurses received an email and contacted the patients within 2 working days. See Multimedia Appendices 1 and 4.

### Outcome Measures

#### Use of the COPD App

Use of the COPD app is measured with log data. Use is reported as the number and percentage of patients and the number of times, described as page clicks, the app and the information items were opened. The number of times the Lung Attack Action Plan, contact request, and CCQ questionnaires were used is described with absolute and relative numbers.

#### Patient Satisfaction

Patients completed questionnaires about satisfaction with app use, the information provided, and user-friendliness. This is assessed on a 7-point scale (1: totally disagree to 7: totally agree). Patients were also asked about their overall satisfaction on a scale of 1 to 10 (1: not satisfied at all to 10: very satisfied). See Multimedia Appendix 5 for the questionnaire.

#### Self-management

The Partners in Health (PIH) scale was used to measure self-management [48,49]. The PIH is a 12-item scale, and the Dutch version consists of 2 subscales: (1) knowledge and coping and (2) recognition and management of symptoms, adherence to treatment. The Cronbach alphas of the subscales were .80 (knowledge and coping) and .72 (recognition and management of symptoms, adherence to treatment). The correlation between the subscales was 0.43. The items are rated on a 9-point Likert scale (0: low self-management and 8: high self-management). The first subscale consists of 7 items, and the second subscale consists of 5 items [49]. The total score for both subscales was calculated by taking the sum of the respective items.

#### Expectations and Experiences With the COPD App

Questionnaires covering constructs of the Unified Theory of Acceptance and Use of Technology (UTAUT) [50] model were used to measure expectations (baseline) and experiences (weeks 8 and 20) with using the COPD app. The UTAUT consists of 4 constructs that influence behavioral intention and behavior: (1) performance expectancy, (2) effort expectancy, (3) social influence, and (4) facilitating conditions. A total of 8 questions were rated on a 7-point scale (1: totally disagree to 7: totally agree). See Multimedia Appendix 6 for the questionnaires.

#### Satisfaction of Nurses

After all patients were included and completed the 8-week self-management program, we asked involved pulmonary nurses about their experience with the COPD app, video consultation, experience with monitoring the CCQ scores, and their satisfaction with for example efficiency and time investment.

#### Hospital Readmissions

A hospital readmission was defined as admission for at least 24 hours. The number of hospital admissions was obtained from the electronic medical record (EMR) after 30 days, 8 weeks, and 20 weeks. This was compared with the readmission rate from the previous year, November 2017 to November 2018.

#### Other Outcomes

Patients’ age, Global Initiative for Chronic Obstructive Lung Disease (GOLD) stage, and comorbidities were extracted from the EMR. Their marital status, education, internet use, smartphone or tablet skills, and need for support using a smartphone or tablet were assessed using a questionnaire.

### Data Collection

Use was assessed using log data, extracted from the app software, after 8 and 20 weeks. Patients completed a baseline questionnaire during hospital admission, covering aspects of self-management (PIH), expectations with the COPD app, internet use, smartphone or tablet skills, and sociodemographics. After 8 weeks and 20 weeks, a questionnaire was sent on self-management, experiences with the app, and (overall) satisfaction. After 30 days, 8 weeks, and 20 weeks, the readmission rate was assessed, and data were extracted from the EMR. See Table 1 for an overview of the outcomes and measurement time points.

**Table 1:** Outcomes and measurement time points.

Outcome	Measurement instrument	Baseline	30 days	Week 8	Week 20
Use of the COPD app	Log data	—^a^	—	—	—
Self-management	PIH^b^ scale	●^c^	X^d^	●	●
Expectations with the COPD^e^ app	Questionnaire (UTAUT^f^ constructs)	●	X	X	X
Experiences with the COPD app	Questionnaire (UTAUT constructs)	X	X	●	●
Satisfaction (functionalities of the COPD app)	Questionnaire	X	X	●	X
Overall satisfaction	10-point scale	X	X	●	●
Readmissions	EMR^g^	X	●	●	●

^a^—: Weekly assessment from baseline until 20 weeks.

^b^PIH: Partners in Health.

^c^Outcome measurement.

^d^No outcome measurement.

^e^COPD: chronic obstructive pulmonary disease.

^f^UTAUT: Unified Theory of Acceptance and Use of Technology.

^g^EMR: electronic medical record.

### Statistical Analysis

Data analysis was performed using IBM SPSS V22.0. Descriptive statistics were used to report the baseline characteristics, app use, expectations and experiences, satisfaction, and number of readmissions. Changes in self-management over time were analyzed using a linear mixed model. Using a linear mixed model allowed for the inclusion of cases with missing data. The relation between app use and self-management was analyzed using linear regression. Normally distributed variables were reported as mean and standard deviation, and non-normally distributed data were reported with medians and interquartile ranges (25th-75th percentiles).

### Approval and Ethical Considerations

The study was approved by the local ethical committee *Commissie Mensgebonden Onderzoek Arnhem–Nijmegen*.

## Results

### Pilot Testing

A total of 6 patients participated in the pilot testing of a prototype of the COPD app: 3 men and 3 women. The age range was 58-78 years. A total of 4 patients used the internet (almost) every day and 2 patients (less than) 1 day per week. Moreover, 3 patients used a smartphone or tablet (almost) every day, 1 patient multiple days per week, and 2 patients never. Furthermore, 3 out of 6 patients perceived their smartphone or tablet skills not good or not bad, 1 bad, and 1 good. In addition, 3 (out of 6) patients did not miss information items in the COPD app.

The information was categorized per day in the prototype, meaning that a new information item was presented daily. During the assignments and observations, we found that it was not easy for patients to find information because the timeline was very long. A total of 4 (out of 6) patients preferred to receive all information items in 1 overview, ordered by information category (eg, nutrition). On the basis of the findings, we categorized the information per category (eg, nutrition, physical activity) instead of per day. To increase ease of use, the 8-week program was classified per week instead of per day. Patients’ opinion about the frequency of receiving a notification varied. Therefore, we decided to send a weekly reminder about the Lung Attack Action Plan and a reminder to fill out the weekly CCQ questionnaire.

### Feasibility Study—Patient Recruitment

**Figure 1:**
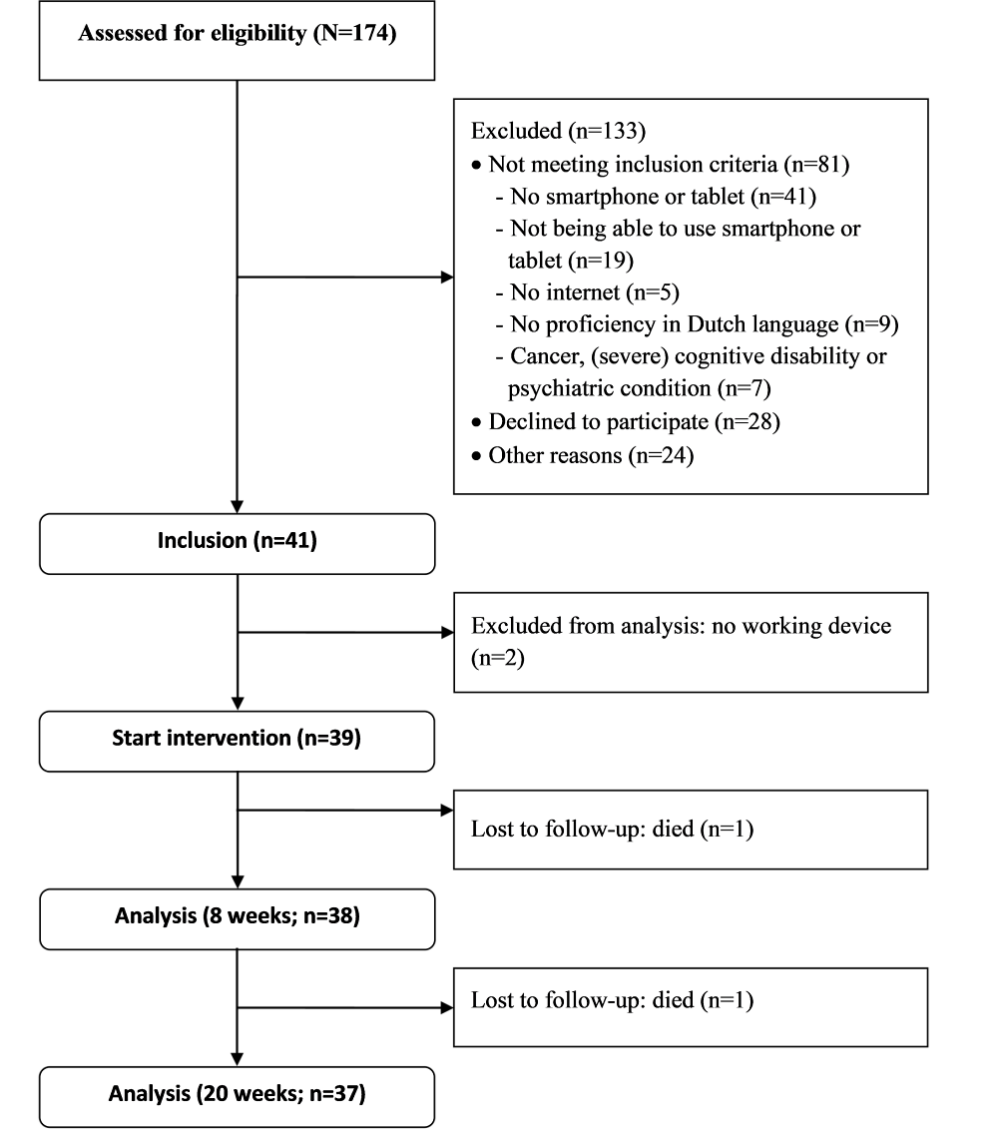
Flow diagram.

Inclusion took place from November 19, 2018, to December 13, 2019. A total of 174 patients were assessed for eligibility. Moreover, 81 patients did not meet the inclusion criteria because they had no access to a smartphone or tablet (n=41), were not able to use a smartphone or tablet (n=19), no working internet connection (n=5), no proficiency in Dutch language (n=9), cancer, (severe) cognitive disability or psychiatric condition (n=7), or other reasons (n=24 eg, hospital admissions were too short, unclear diagnosis, or no reason was reported). In total, 28 patients declined to participate. Moreover, 2 patients signed the informed consent form, but they were excluded because the COPD app could not be installed on their smartphone or tablet. In total, 39 patients started the intervention. One patient died during the first 8 weeks, and 1 patient died before 20 weeks. Therefore, 39 patients were included in the analysis until 8 weeks, 38 patients were included in the analysis at week 8 and from week 8 to week 20, and 37 patients were included in the analysis at 20 weeks (Figure 1).

### Baseline Characteristics

The baseline characteristics of the population included in the feasibility study are presented in Table 2.

**Table 2:** Baseline characteristics (N=39).

Baseline characteristics		Patients
**Gender, n (%)**		
	Women	30 (77)
	Men	9 (23)
Age (years), mean (SD)		62.2 (6.7)
**Severity classification, n (%)^a^**		
	Moderate (GOLD stage 2)	7 (18)
	Very severe (GOLD stage 3+4)	32 (82)
Living with a partner, n (%)^a^		25 (68)
Having children, n (%)^a^		34 (92)
Children living at home, n (%)^a^		10 (30)
**Education, n (%)^a^**		
	Low (primary school)	12 (32)
	Middle (high school or vocational education)	22 (60)
	High (higher vocational education or university)	3 (8)
**Comorbidities, n (%)^a^**		
	Hypertension	7 (18)
	Depression	3 (8)
	Diabetes	2 (5)
	Asthma	2 (5)
	Heart disease	2 (5)
	Reuma	2 (5)
**Internet use (duration), n (%)^a,b^**		
	<6 months	2 (5)
	6 months to 2 years	2 (5)
	>2 years	2 (5)
	>3 years	31 (84)
**Frequency of internet use, n (%)^a^**		
	Almost every day	32 (86)
	Multiple days a week	3 (8)
	About 1 day a week	1 (3)
	Never	1 (3)
**Smartphone or tablet skills, n (%)^a,b^**		
	Bad and/or very bad	7 (19)
	Not good and/or not bad	16 (44)
	Good and/or very good	13 (36)
Expects to need help with smartphone or tablet use, n (%)^a^		21 (58)

^a^Reported as valid percentage.

^b^Does not add up to 100% because of rounding.

### Use

The use of the COPD app, questionnaires, and consultations is described in more detail below and is presented in Table 3.

**Table 3:** Overview of the use of the chronic obstructive pulmonary disease app functionalities (N=39).

Functionalities			Patients, n (%)
**COPD^a^ app use**			
	Week 1	39 (100)	
	Week 2	33 (85)	
	Week 3	32 (82)	
	Week 4-8	31 (79)	
**CCQ^b^ questionnaires**			
	9 weekly CCQ questionnaires completed	29 (74)	
	8 weekly CCQ questionnaires completed	3 (8)	
	7 weekly CCQ questionnaires completed	4 (10)	
	<7 weekly CCQ questionnaires completed	3 (8)	
**HADS^c^**			
	Week 1: questionnaire completed	35 (90)	
	Week 8: questionnaire completed	33 (85)	
**Video consultation (week 4)**			
	Video consultation	17 (44)	
	Telephonic consultation	13 (33)	
	No video consultation	9 (23)	
**Face-to-face consultation (week 8)**			
	Face-to-face consultation	27 (69)	
	Telephonic consultation	1 (2)	
	No face-to-face consultation (canceled)	11 (28)	
**Lung Attack Action Plan (week 1-8)**			
	Use Lung Attack Action Plan and request for contact	9 (23)	
	Contact with a nurse as a result of the use of the Lung Attack Action Plan	9 (100)	
**Contact page (week 1-8)**			
	Request for contact using contact page	3 (8)	
	Contact with a nurse as a result of the use of the contact page	3 (100)	

^a^COPD: chronic obstructive pulmonary disease.

^b^CCQ: Clinical COPD Questionnaire.

^c^HADS: Hospital Anxiety and Depression Scale.

#### COPD App

The use of the COPD app varied widely across patients. The app was opened most often during the first week (median 6.0; IQR 3.5-10.0). However, use decreased over time. The app was opened by the majority of patients during the first 8 weeks, varying from 100% (39/39) in the first week to 79% (31/39) in week 8. Patients read information most frequently during the first week, especially regarding the functionalities in the COPD app (27/39, 69%), physical activity (24/39, 62%), the condition COPD, nutrition, and the Lung Attack Action Plan (22/39, 56%). See Multimedia Appendix 7 for detailed information.

#### Questionnaires (CCQ and HADS) and Monitoring

In total, 29 patients filled out all the weekly CCQ questionnaires (in total 9 times including baseline), 3 answered the CCQ during 8 weeks, 4 answered the CCQ during 7 weeks, 1 answered the CCQ during 6 weeks, and 2 answered the CCQ during 2 weeks. A total of 35 patients filled out the HADS in week 1 (after discharge) and 33 after 8 weeks. Two patients reported that they did not want to fill out the questionnaires anymore during the study, and 1 patient died 7 weeks after discharge. The monitoring of the scores was used inconsistently, and therefore, the results do not offer a meaningful contribution.

#### Consultations

A total of 17 patients attended the planned video consultation 4 weeks after discharge. For 13 other patients, this was replaced by a telephonic consultation because of problems with the video consultation system (eg, technical issues or lack of skills from nurses or patients); 2 patients did not want a video consultation; 1 patient visited the hospital instead; 1 patient’s consultation was canceled because of hospital readmission; 1 patient left the digital waiting room because the nurse was too late; 1 patient was not available; and for 3 patients, a reason for cancelation was not reported.

A total of 27 patients attended their face-to-face consultation after approximately 8 weeks. For 11 other patients, the appointment was canceled because patients did not show up (n=5), because of readmission (n=3), two patients canceled the appointment, and 1 patient died. For 1 patient, this consultation was replaced by a telephonic consultation because the patients did not feel fit enough to come to the hospital.

In total, additional contact with a nurse was requested 19 times. A total of 9 patients used the Lung Attack Action Plan 15 times (13 times code yellow and 2 times orange), and 3 patients used the contact form 4 times to request contact with a nurse. See Multimedia Appendix 7 for more details on the use of the Lung Attack Action Plan.

### Satisfaction

The COPD app was rated, on a scale of 1 to 10 (1: not satisfied at all to 10: very satisfied), with a 7.7 (SD 1.7) after 8 weeks and 7.0 (SD 2.4) after 20 weeks. Patients thought the app was easy to use and well-structured (26/28, 93%). Almost all patients reported that the Lung Attack Action Plan was easy to find (27/28, 96%) and easy to use (25/27, 93%), and more than half of the patients thought it actually helped them (18/27, 67%). The majority of patients also thought that the information was understandable (27/29, 93%), and all the patients (29/29, 100%) were satisfied with the information about nutrition. According to 33% (9/27) of patients, too much information was available in the COPD app. The majority of patients were satisfied with the video consultations (18/23, 78%) and thought it saved them time (19/29, 66%). See Multimedia Appendix 8 for more detailed information.

### Self-management

Knowledge and coping increased significantly over time (*P*=.04). However, there was no significant change in the recognition and management of symptoms (*P=*.14). See Multimedia Appendix 9. 

### Relation Between App Use and Self-management

No relation was not found between use of the app, the number of times the app was opened (mean page clicks during week 1-8), and the self-management elements *knowledge and coping* (*P*=.75) and *recognition management and adherence* (*P*=.92).

### Expectations and Experiences With the COPD App (Technology Acceptance)

Patients’ expectations with the COPD app were relatively high. However, only 2 aspects improved over time. After using the app, more patients thought that it takes no effort to use it and that they had enough skills to use it. However, most aspects related to receiving support using the app decreased over time. See Multimedia Appendix 10 for more detailed information.

### Satisfaction of Nurses

The use of the COPD app and monitoring of the weekly questionnaires were evaluated with 3 nurses. They rated the COPD app, on a scale of 1 to 10 (1: not satisfied at all to 10: very satisfied), on average with a 6.3 (SD 1.2) Most of them were satisfied with the app (2/3, 67%) and the information provided (2/3, 67%) and thought that better care was provided using the COPD app (2/3, 67%). However, use of the COPD app did not save time (3/3, 100%). They received a lot of questions from patients (3/3, 100%), and they mentioned that it took them a lot of time to explain it and answer questions (2/3, 67%). They also reported:

Unfortunately not applicable for our target population, the app is good.

How simple it seemed to use, how difficult it appeared to be for patients.

Only 1 nurse would recommend the COPD app to more patients. The nurses would not recommend it to their colleagues.

The nurses were less satisfied with monitoring the results of the questionnaires and rated this with a 5.3 (SD 0.58), on a scale of 1 to 10 (1: not satisfied at all to 10: very satisfied). Only 1 nurse thought that monitoring the results of the questionnaires fitted well in their work process. They commented:

Plan more time for nurses to monitor the questionnaires.

It is often unclear for patients what they have to fill out. Sometimes patients were surprised when they got a call, because they felt good.

The nurses were less satisfied with the video consultations and mentioned the following:

This was very difficult, very unclear for patients, took a lot of time and often a telephonic consultation was needed.

Many patients did not understand how to start a video consultation.

### Hospital Readmissions

In total, 39 patients were included in the study. A total of 12 patients (12/39, 31%) were readmitted 22 times during the study period (20 weeks), of which 5 patients (5/39, 13%) were readmitted 1 time in the first 30 days. Within 8 weeks, 8 patients (8/39, 21%) were readmitted 11 times. In the total study period (until 20 weeks), there were 22 readmissions for 12 patients (12/39, 31%). The main reasons for readmissions was COPD exacerbations, and 1 time it was due to a patient’s home situation.

In the year preceding the study, from November 2017 to November 2018, 340 patients were admitted 478 times to the hospital. In total, 48 patients (48/340, 14.1%) were readmitted 77 times within 30 days. There were 103 readmissions within 8 weeks for 61 patients (61/340, 17.9%), and 74 patients (74/340, 21.8%) were readmitted 129 times within 20 weeks.

## Discussion

### Principal Findings

In this study, a mobile self-management app for high-risk patients with COPD was evaluated in daily clinical practice. The COPD app was opened most often in the first week (median 6.0; IQR 3.5-10.0), but its use decreased over time (median 2.0; IQR 1.0-3.5 in week 8). Information, especially on physical activity (24/39, 62%), was read most often during the first week. The self-management element *knowledge and coping* increased significantly over time (*P*=.04), but a relation with app use was not found (*P=.*75). No significant change was found in *recognition and management of symptoms, adherence to treatment* (*P*=.14), or in relation with app use (*P*=. 92). Patients rated the COPD app on average with a 7.7 (SD 1.7) and nurses with a 6.3 (SD 1.2). Preliminary evidence about readmission rate showed that 13% (5/39) of patients were readmitted within 30 days, 21% (8/39) within 8 weeks, and 31% (12/39) within 20 weeks compared with 14.1% (48/340), 17.9% (61/340), and 21.8% (74/340), respectively, in a preresearch cohort.

### Comparison With Prior Work

The use of mobile apps itself is not applicable to all patients [51,52]. In total, 37.4% (65/174) of all patients in our study had to be excluded because of lack of access to a mobile device or internet or skills to use it. This is in line with other findings of mHealth use in patients with COPD, in which only a minority owned a smartphone (23%) [53]. Technical issues and low compliance are recognized issues for digital interventions [54], and digital literacy among patients with COPD remains a challenge [52]. As a result of the pilot testing, the app we implemented was already simplified. However, digital literacy may still have been an issue during this study. Therefore, ease of use seems to be an essential element in digital interventions for this patient population [20,27]. A total of 16.1% (28/174) of those possibly qualifying declined to participate, among other things, because it was too much of a burden or effort at the time. Patients may have experienced high levels of distress after experiencing an exacerbation [55], and therefore, they may be less willing to engage in a self-management intervention [38]. Therefore, these interventions are not applicable to all patients who are recently discharged from the hospital [38], as they may still feel (too) sick and/or are not able to focus on the intervention [34]. This emphasizes the importance of timing [39] and tailoring [56] an intervention.

Until now, the effects of self-management interventions on patients recently discharged from the hospital were scarcely evaluated [38] in combination with mobile apps. The direct effects [57] of app supported self-management and health interventions, for example, technology acceptance, self-management, and patients’ and nurses’ satisfaction are relevant for use in clinical practice. We found that the app was especially used during the first week after discharge. The Lung Attack Action Plan (9/39, 23%) and request for contact using the contact page (3/39, 8%) were used to a limited extent. However, the majority (29/39, 74%) completed the weekly CCQ questionnaires during the whole intervention period and the HADS in week 8 (33/39, 85%). Patients received frequent reminders by email, in the app and sometimes from nurses, to complete the questionnaires. The use of the COPD app and the Lung Attack Action Plan was more optional, rather at patients’ own initiative. Receiving feedback can be important [56], and this may explain that the majority of patients completed the questionnaires, but that the use of the COPD app decreased over time. Low frequency of use can also be due to lack of self-management or technological skills [56].

Social support is seen as a facilitator for use [32,52]. The majority of the patients (28/37, 76%) expected to receive enough help using the COPD app. However, only 57% (17/30) of the patients indicated that they had received enough help (Multimedia Appendix 10). Tailored education can also facilitate use [52], but in this COPD app, only the medication overview was really personalized. Although the information items were aimed at high-risk patients with COPD, the information was generic. This might have contributed to the decrease in use. Tailored interventions [56], support [30], and patient engagement during development and implementation [56,58] may be beneficial for improved use.

A positive effect was found on knowledge and coping, which may partly be explained by the selection criteria for this study, as patients with cognitive disability and lack of skills with a mobile device were excluded. In addition, the provision of timely information using a mobile device can positively influence knowledge [59]. Self-management can also be enhanced by involving patients’ partners, enhancing self-efficacy, and support from health care professionals [30]. Although positive results on hospital readmissions were found in previous studies [6,18], these findings were inconsistent [15,28,60], which could be due to high methodological heterogeneity [16,19]. In our study, no large difference was observed, possibly due to low numbers. It would be interesting to verify the element of selection bias in view of the large percentage of patients that were excluded from this population.

Patients were satisfied with the COPD app, user-friendliness, and information. However, nurses addressed some concerns, for example, the increased workload and (lack of) integration in the work process. It is common that the degree of satisfaction between patients and health care professionals can differ. In general, patients report more favorable outcomes because mobile interventions are often provided as an extra service in addition to their usual care. For that same reason, health care professionals are generally less satisfied, especially because they often see it as an increase in workload [61]. The nurses in our study addressed concerns about the monitoring of the results of the questionnaires because they experienced a lack of integration in their work processes. Often a common pattern with the introduction of new innovations, this intervention was an addition to their current activities. Another reason might be that nurses had to work with different information technology systems that were not connected to the EMR. Lack of interoperability can be a barrier [58] for use, and this might explain the lack of monitoring of the first phase of the study. This improved after they received the scores in person by email. Health care professionals’ adoption is essential to ensure success; therefore, they should be involved in the development and implementation process [56].

COPD management requires a multidisciplinary approach that is fragmented [24], and this approach is often not sufficiently supported by information technology [62]. Therefore, future research should focus on self-management interventions with a multidisciplinary approach tailored to individual patients recently discharged from the hospital. Pragmatic trials [63] can be used to determine, at a more rapid pace, which elements of self-management interventions are effective for which subgroups of patients with COPD recently discharged and which characteristics of mHealth solutions are adopted by both patients and health care professionals. Subsequently, a larger controlled study specifically involving this frail subgroup of patients should focus on the effects on clinical outcomes and hospital services use (eg, readmissions).

### Limitations

Due to accrual issues, especially related to device availability and internet access, the COPD app was evaluated in a small sample, so we could not reach the power originally calculated for this trial. In addition, nurses found it difficult to comply with the contacting rules, so there were inconsistencies in the follow-up monitoring using the CCQ questionnaires. Some patients were only contacted a limited number of times when they had a high score on the CCQ questionnaire. After approximately 20 patients, we decided to send nurses a notification by email with the scores, and they were asked to take up contact (if necessary). As a consequence of the team setting, only 3 nurses were involved in this study, and we have to be careful about the related outcomes. Preliminary evidence on readmission rates was provided based on an earlier cohort, but this was not a matched exercise. Therefore, definitive conclusions on this aspect cannot be drawn.

### Conclusions

The integration and use of a mobile self-management app for recently discharged patients with COPD in clinical practice is affected by multiple factors and is only feasible for a relatively small number of patients after hospital discharge. Patients were very positive about the COPD app; however, its use decreased over time. The findings of this study showed a significant positive change in the self-management element knowledge and coping. Nurses expressed concerns about integration in their work processes and increased workload. Tailored interventions, patient support, and active adoption by professionals are important elements to ensure successful mHealth interventions. Therefore, future research on digital self-management interventions in clinical practice should focus on including more difficult subgroups of target populations, on a multidisciplinary approach, on technology-related aspects (such as acceptability), and on finetuning its adoption in clinical pathways.

## Appendix

Multimedia Appendix 1 Chronic obstructive pulmonary disease (COPD) app.

Multimedia Appendix 2 Timeline (English translation).

Multimedia Appendix 3 Information page (English translation).

Multimedia Appendix 4 Contact page (English translation).

Multimedia Appendix 5 Questionnaire: Patient Satisfaction.

Multimedia Appendix 6 Questionnaire: Expectations and Experiences with the COPD app.

Multimedia Appendix 7 Use of the chronic obstructive pulmonary disease (COPD) app.

Multimedia Appendix 8 Patient satisfaction.

Multimedia Appendix 9 Self-management.

Multimedia Appendix 10 Expectations of and experiences with the chronic obstructive pulmonary disease (COPD) app.

## Abbreviations

CCQ: Clinical COPD Questionnaire
COPD: chronic obstructive pulmonary disease
EMR: electronic medical record
HADS: Hospital Anxiety and Depression Scale
mHealth: mobile health
PIH: Partners in Health
UTAUT: Unified Theory of Acceptance and Use of Technology

## References

[1] World Health Organization (2017). 2017. Chronic obstructive pulmonary disease (COPD).

[2] Rijksinstituut oor Volksgezondheid en Milieu (2018). COPD.

[3] Global Initiative for Chronic Obstructive Lung Disease (2020). 2020. Global Strategy for the Diagnosis, Management, and Prevention of Chronic Obstructive Pulmonary Disease.

[4] Vogelmeier CF, Criner GJ, Martinez FJ, Anzueto A, Barnes PJ, Bourbeau J, Celli BR, Chen R, Decramer M, Fabbri LM, Frith P, Halpin DMG, López Varela M Victorina, Nishimura M, Roche N, Rodriguez-Roisin R, Sin DD, Singh D, Stockley R, Vestbo J, Wedzicha JA, Agusti A (2017). Global Strategy for the Diagnosis, Management, and Prevention of Chronic Obstructive Lung Disease 2017 Report: GOLD Executive Summary. Eur Respir J.

[5] (2017). From the Global Strategy for the Diagnosis, Management and Prevention of COPD, Global Initiative for Chronic Obstructive Lung Disease (GOLD). GOLD 2017.

[6] McLean S, Nurmatov U, Liu JL, Pagliari C, Car J, Sheikh A (2011). Telehealthcare for chronic obstructive pulmonary disease. Cochrane Database Syst Rev.

[7] Garcia-Aymerich J, Farrero E, Félez M A, Izquierdo J, Marrades RM, Antó J M, Estudi del Factors de Risc d'Agudització de la MPOC investigators (2003). Risk factors of readmission to hospital for a COPD exacerbation: a prospective study. Thorax.

[8] Toy EL, Gallagher KF, Stanley EL, Swensen AR, Duh MS (2010). The economic impact of exacerbations of chronic obstructive pulmonary disease and exacerbation definition: a review. COPD.

[9] Zwerink M, Brusse-Keizer M, van der Valk PDLPM, Zielhuis GA, Monninkhof EM, van der Palen J, Frith PA, Effing T (2014). Self management for patients with chronic obstructive pulmonary disease. Cochrane Database Syst Rev.

[10] Lenferink A, Brusse-Keizer M, van der Valk PDLPM, Frith PA, Zwerink M, Monninkhof EM, van der Palen J, Effing TW (2017). Self-management interventions including action plans for exacerbations versus usual care in patients with chronic obstructive pulmonary disease. Cochrane Database Syst Rev.

[11] Wang T, Tan JY, Xiao LD, Deng R (2017). Effectiveness of disease-specific self-management education on health outcomes in patients with chronic obstructive pulmonary disease: An updated systematic review and meta-analysis. Patient Educ Couns.

[12] Effing TW, Vercoulen JH, Bourbeau J, Trappenburg J, Lenferink A, Cafarella P, Coultas D, Meek P, van der Valk P, Bischoff EW, Bucknall C, Dewan NA, Early F, Fan V, Frith P, Janssen DJ, Mitchell K, Morgan M, Nici L, Patel I, Walters H, Rice KL, Singh S, Zuwallack R, Benzo R, Goldstein R, Partridge MR, van der Palen J (2016). Definition of a COPD self-management intervention: International Expert Group consensus. Eur Respir J.

[13] Bourbeau J, van der Palen J (2009). Promoting effective self-management programmes to improve COPD. Eur Respir J.

[14] Effing TW, Bourbeau J, Vercoulen J, Apter AJ, Coultas D, Meek P, van der Valk P, Partridge MR, van der Palen J (2012). Self-management programmes for COPD: moving forward. Chron Respir Dis.

[15] McCabe C, McCann M, Brady AM (2017). Computer and mobile technology interventions for self-management in chronic obstructive pulmonary disease. Cochrane Database Syst Rev.

[16] Alwashmi M, Hawboldt J, Davis E, Marra C, Gamble JM, Abu Ashour W (2016). The Effect of Smartphone Interventions on Patients With Chronic Obstructive Pulmonary Disease Exacerbations: A Systematic Review and Meta-Analysis. JMIR Mhealth Uhealth.

[17] Shaw G, Whelan ME, Armitage LC, Roberts N, Farmer AJ (2020). Are COPD self-management mobile applications effective? A systematic review and meta-analysis. NPJ Prim Care Respir Med.

[18] Yang F, Wang Y, Yang C, Hu H, Xiong Z (2018). Mobile health applications in self-management of patients with chronic obstructive pulmonary disease: a systematic review and meta-analysis of their efficacy. BMC Pulm Med.

[19] Lundell S, Holmner Å, Rehn B, Nyberg A, Wadell K (2015). Telehealthcare in COPD: a systematic review and meta-analysis on physical outcomes and dyspnea. Respir Med.

[20] Williams V, Price J, Hardinge M, Tarassenko L, Farmer A (2014). Using a mobile health application to support self-management in COPD: a qualitative study. Br J Gen Pract.

[21] Park SK, Bang CH, Lee SH (2020). Evaluating the effect of a smartphone app-based self-management program for people with COPD: A randomized controlled trial. Appl Nurs Res.

[22] Effing T, Monninkhof EM, van der Valk PD, van der Palen J, van Herwaarden CL, Partidge MR, Walters EH, Zielhuis GA (2007). Self-management education for patients with chronic obstructive pulmonary disease. Cochrane Database Syst Rev.

[23] (2019). GOLD 2019. Global initiative for chronic obstructive lung disease.

[24] Sobnath DD, Philip N, Kayyali R, Nabhani-Gebara S, Pierscionek B, Vaes AW, Spruit MA, Kaimakamis E (2017). Features of a Mobile Support App for Patients With Chronic Obstructive Pulmonary Disease: Literature Review and Current Applications. JMIR Mhealth Uhealth.

[25] Vorrink SN, Kort HS, Troosters T, Lammers JW (2016). A Mobile Phone App to Stimulate Daily Physical Activity in Patients with Chronic Obstructive Pulmonary Disease: Development, Feasibility, and Pilot Studies. JMIR Mhealth Uhealth.

[26] Liu WT, Wang CH, Lin HC, Lin SM, Lee KY, Lo YL, Hung SH, Chang YM, Chung KF, Kuo HP (2008). Efficacy of a cell phone-based exercise programme for COPD. Eur Respir J.

[27] Chau JP, Lee DT, Yu DS, Chow AY, Yu WC, Chair SY, Lai AS, Chick YL (2012). A feasibility study to investigate the acceptability and potential effectiveness of a telecare service for older people with chronic obstructive pulmonary disease. Int J Med Inform.

[28] Pinnock H, Hanley J, McCloughan L, Todd A, Krishan A, Lewis S, Stoddart A, van der Pol M, MacNee W, Sheikh A, Pagliari C, McKinstry B (2013). Effectiveness of telemonitoring integrated into existing clinical services on hospital admission for exacerbation of chronic obstructive pulmonary disease: researcher blind, multicentre, randomised controlled trial. BMJ.

[29] Pedone C, Lelli D (2015). Systematic review of telemonitoring in COPD: an update. Pneumonol Alergol Pol.

[30] Warwick M, Gallagher R, Chenoweth L, Stein-Parbury J (2010). Self-management and symptom monitoring among older adults with chronic obstructive pulmonary disease. J Adv Nurs.

[31] Tabak M, Brusse-Keizer M, van der Valk P, Hermens H, Vollenbroek-Hutten M (2014). A telehealth program for self-management of COPD exacerbations and promotion of an active lifestyle: a pilot randomized controlled trial. COPD.

[32] Sun N, Rau PL (2015). The acceptance of personal health devices among patients with chronic conditions. Int J Med Inform.

[33] Peek ST, Wouters EJ, van Hoof J, Luijkx KG, Boeije HR, Vrijhoef HJM (2014). Factors influencing acceptance of technology for aging in place: a systematic review. Int J Med Inform.

[34] Orme MW, Weedon AE, Saukko PM, Esliger DW, Morgan MD, Steiner MC, Downey JW, Sherar LB, Singh SJ (2018). Findings of the Chronic Obstructive Pulmonary Disease-Sitting and Exacerbations Trial (COPD-SEAT) in Reducing Sedentary Time Using Wearable and Mobile Technologies With Educational Support: Randomized Controlled Feasibility Trial. JMIR Mhealth Uhealth.

[35] Vitacca M, Montini A, Comini L (2018). How will telemedicine change clinical practice in chronic obstructive pulmonary disease?. Ther Adv Respir Dis.

[36] Jonkman NH, Westland H, Trappenburg JC, Groenwold RH, Bischoff EW, Bourbeau J, Bucknall CE, Coultas D, Effing TW, Epton MJ, Gallefoss F, Garcia-Aymerich J, Lloyd SM, Monninkhof EM, Nguyen HQ, van der Palen J, Rice KL, Sedeno M, Taylor SJ, Troosters T, Zwar NA, Hoes AW, Schuurmans MJ (2016). Do self-management interventions in COPD patients work and which patients benefit most? An individual patient data meta-analysis. Int J Chron Obstruct Pulmon Dis.

[37] Hallensleben C, van Luenen S, Rolink E, Ossebaard HC, Chavannes NH (2019). eHealth for people with COPD in the Netherlands: a scoping review. COPD.

[38] Majothi S, Jolly K, Heneghan NR, Price MJ, Riley RD, Turner AM, Bayliss SE, Moore DJ, Singh SJ, Adab P, Fitzmaurice DA, Jordan RE (2015). Supported self-management for patients with COPD who have recently been discharged from hospital: a systematic review and meta-analysis. Int J Chron Obstruct Pulmon Dis.

[39] Harrison SL, Apps L, Singh SJ, Steiner MC, Morgan MD, Robertson N (2014). 'Consumed by breathing' - a critical interpretive meta-synthesis of the qualitative literature. Chronic Illn.

[40] Patient Journey App. Patient Journey App.

[41] FaceTalk. FaceTalk.

[42] (2018). Rijnstate Zorgapp. Rijnstate Zorgapp, Google Play Store.

[43] (2018). Rijnstate Zorgapp, Apple App store.

[44] Longaanval actieplan. Longfonds.

[45] Zigmond AS, Snaith RP (1983). The hospital anxiety and depression scale. Acta Psychiatr Scand.

[46] Spinhoven P, Ormel J, Sloekers PP, Kempen GI, Speckens AE, van Hemert AM (1997). A validation study of the Hospital Anxiety and Depression Scale (HADS) in different groups of Dutch subjects. Psychol Med.

[47] van der Molen T, Willemse BW, Schokker S, ten Hacken NH, Postma DS, Juniper EF (2003). Development, validity and responsiveness of the Clinical COPD Questionnaire. Health Qual Life Outcomes.

[48] Petkov J, Harvey P, Battersby M (2010). The internal consistency and construct validity of the partners in health scale: validation of a patient rated chronic condition self-management measure. Qual Life Res.

[49] Lenferink A, Effing T, Harvey P, Battersby M, Frith P, van Beurden W, van der Palen J, Paap MC (2016). Construct Validity of the Dutch Version of the 12-Item Partners in Health Scale: Measuring Patient Self-Management Behaviour and Knowledge in Patients with Chronic Obstructive Pulmonary Disease. PLoS One.

[50] Venkatesh V, Morris MG, Davis GB, Davis FD (2003). User Acceptance of Information Technology: Toward a Unified View. MIS Quarterly.

[51] Sanders C, Rogers A, Bowen R, Bower P, Hirani S, Cartwright M, Fitzpatrick R, Knapp M, Barlow J, Hendy J, Chrysanthaki T, Bardsley M, Newman SP (2012). Exploring barriers to participation and adoption of telehealth and telecare within the Whole System Demonstrator trial: a qualitative study. BMC Health Serv Res.

[52] Slevin P, Kessie T, Cullen J, Butler MW, Donnelly SC, Caulfield B (2019). A qualitative study of chronic obstructive pulmonary disease patient perceptions of the barriers and facilitators to adopting digital health technology. Digit Health.

[53] Alwashmi MF, Fitzpatrick B, Farrell J, Gamble JM, Davis E, Nguyen HV, Farrell G, Hawboldt J (2020). Perceptions of Patients Regarding Mobile Health Interventions for the Management of Chronic Obstructive Pulmonary Disease: Mixed Methods Study. JMIR Mhealth Uhealth.

[54] Ding H, Fatehi F, Maiorana A, Bashi N, Hu W, Edwards I (2019). Digital health for COPD care: the current state of play. J Thorac Dis.

[55] Wu RC, Ginsburg S, Son T, Gershon AS (2019). Using wearables and self-management apps in patients with COPD: a qualitative study. ERJ Open Res.

[56] Korpershoek YJG, Vervoort SCJM, Trappenburg JCA, Schuurmans MJ (2018). Perceptions of patients with chronic obstructive pulmonary disease and their health care providers towards using mHealth for self-management of exacerbations: a qualitative study. BMC Health Serv Res.

[57] Bravo P, Edwards A, Barr PJ, Scholl I, Elwyn G, McAllister M, Cochrane Healthcare Quality Research Group, Cardiff University (2015). Conceptualising patient empowerment: a mixed methods study. BMC Health Serv Res.

[58] Sleurs K, Seys SF, Bousquet J, Fokkens WJ, Gorris S, Pugin B, Hellings PW (2019). Mobile health tools for the management of chronic respiratory diseases. Allergy.

[59] Timmers T, Janssen L, Kool RB, Kremer JA (2020). Educating Patients by Providing Timely Information Using Smartphone and Tablet Apps: Systematic Review. J Med Internet Res.

[60] Sul AR, Lyu DH, Park DA (2020). Effectiveness of telemonitoring versus usual care for chronic obstructive pulmonary disease: A systematic review and meta-analysis. J Telemed Telecare.

[61] Brunton L, Bower P, Sanders C (2015). The Contradictions of Telehealth User Experience in Chronic Obstructive Pulmonary Disease (COPD): A Qualitative Meta-Synthesis. PLoS One.

[62] Kooij L, Groen WG, van Harten WH (2017). The Effectiveness of Information Technology-Supported Shared Care for Patients With Chronic Disease: A Systematic Review. J Med Internet Res.

[63] Peterson ED, Harrington RA (2018). Evaluating Health Technology Through Pragmatic Trials: Novel Approaches to Generate High-Quality Evidence. JAMA.

