# Supplementary material for: Effectiveness of a Mobile Health and Self-Management App for High-Risk Patients With Chronic Obstructive Pulmonary Disease in Daily Clinical Practice: Mixed Methods Evaluation Study
Source: JMIR Mhealth Uhealth. 2021 Feb 4;9(2):e21977. doi: 10.2196/21977 (PMC7892284; doi:10.2196/21977)
Supplement: Multimedia Appendix 2 [file mhealth_v9i2e21977_app2.pdf]

COPD app - Timeline

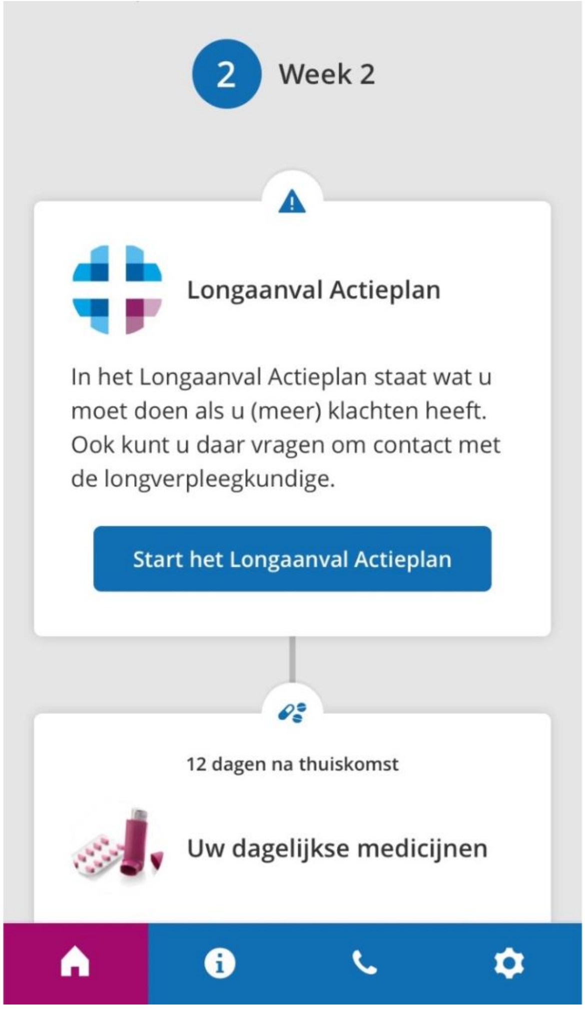

English translation:

Week 2

Lung Attack Action Plan

In the Lung Attack Action Plan you can find what to do if you have (worsening) symptoms. You can also request contact with a pulmonary nurse.

Your daily medication
