# Supplementary material for: Effectiveness of a Mobile Health and Self-Management App for High-Risk Patients With Chronic Obstructive Pulmonary Disease in Daily Clinical Practice: Mixed Methods Evaluation Study
Source: JMIR Mhealth Uhealth. 2021 Feb 4;9(2):e21977. doi: 10.2196/21977 (PMC7892284; doi:10.2196/21977)
Supplement: Multimedia Appendix 3 [file mhealth_v9i2e21977_app3.pdf]

COPD app - Information page

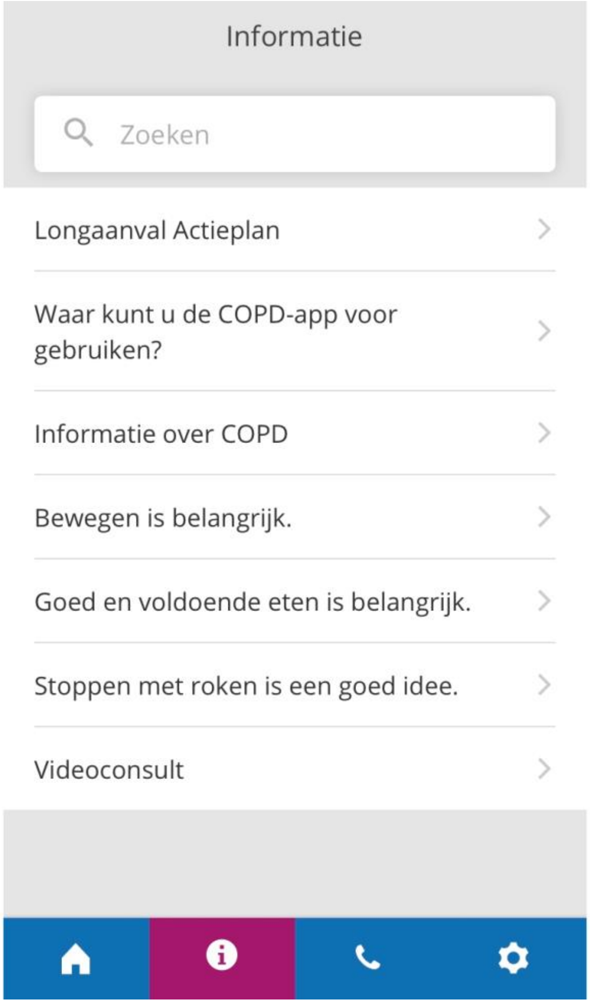

English translation:

Information

Search

Lung Attack Action Plan ›

What to use the COPD app for? ›

Information about COPD ›

Physical activity is important ›

Good and enough nutrition is important ›

Smoking cessation is a good idea ›

Video consultation ›
