# Supplementary material for: Effectiveness of a Mobile Health and Self-Management App for High-Risk Patients With Chronic Obstructive Pulmonary Disease in Daily Clinical Practice: Mixed Methods Evaluation Study
Source: JMIR Mhealth Uhealth. 2021 Feb 4;9(2):e21977. doi: 10.2196/21977 (PMC7892284; doi:10.2196/21977)
Supplement: Multimedia Appendix 4 [file mhealth_v9i2e21977_app4.pdf]

COPD app - Contact page

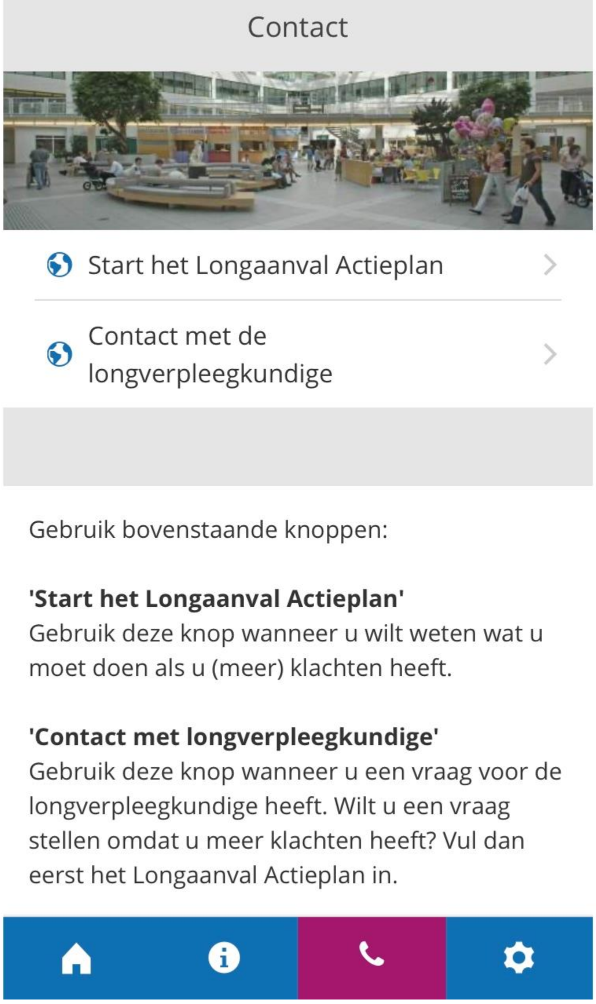

<u>English translation:</u>

Contact

- Start the Lung Attack Action Plan
- Contact with a pulmonary nurse

Use buttons above:

'Start the Lung Attack Action Plan'

Use this button if you want to know what to do when you have (worsening) complaints.

'Contact the pulmonary nurse'

Use this button if you have a question for the pulmonary nurse. Is your question related to worsening complaints? Use the Lung Attack Action Plan first.
