# Supplementary material for: Effectiveness of a Mobile Health and Self-Management App for High-Risk Patients With Chronic Obstructive Pulmonary Disease in Daily Clinical Practice: Mixed Methods Evaluation Study
Source: JMIR Mhealth Uhealth. 2021 Feb 4;9(2):e21977. doi: 10.2196/21977 (PMC7892284; doi:10.2196/21977)
Supplement: Multimedia Appendix 5 [file mhealth_v9i2e21977_app5.pdf]

Patient Satisfaction Questionnaire

Usability (7-point scale, 1: totally disagree to 7: totally agree)

1. Log in to the app is easy
2. The COPD app is well-structured

Lung Attack Action Plan (7-point scale, 1: totally disagree to 7: totally agree)

3. The Lung Attack Action Plan is easy to find in the app
4. The Lung Attack Action Plan is easy to use
5. The Lung Attack Action Plan helped me

Information (7-point scale, 1: totally disagree to 7: totally agree)

6. I prefer to receive my information via video instead of text
7. I am satisfied with the information I received about the condition COPD (for example about functioning of the lungs and lung exacerbations)
8. I am satisfied with my, daily and extra, medication overview in the app
9. I am satisfied with the information about breathing technique(s)
10. I am satisfied with the information about nutrition
11. I am satisfied with the information about physical activity
12. If applicable, I am satisfied with the information about the advantages of smoking cessation
13. There is too much information available in the COPD app
14. I prefer to receive more frequent reminders in the app, regarding new information or questionnaires

15. I missed information about (multiple answers possible):
    - ☐ The condition COPD
    - ☐ Lung exacerbations
    - ☐ Breathing techniques
    - ☐ Nutrition
    - ☐ Physical activity
    - ☐ Smoking
    - ☐ Otherwise, namely:__________________________
    - ☐ I did not miss information

16. In general, how satisfied are you with the COPD app?

Rate from 1 to 10
1 = very unsatisfied
10 = very satisfied

1 2 3 4 5 6 7 8 9 10

17. Do you have suggestions to improve the COPD app?

____

Video consultation (7-point scale, 1: totally disagree to 7: totally agree)

18. I am satisfied with video consultation
19. I could hear and see the nurse clearly during video consultation
20. I had problems using video consultation
21. By using video consultation, I saved time because I did not have to come to the hospital
