# Supplementary material for: Effectiveness of a Mobile Health and Self-Management App for High-Risk Patients With Chronic Obstructive Pulmonary Disease in Daily Clinical Practice: Mixed Methods Evaluation Study
Source: JMIR Mhealth Uhealth. 2021 Feb 4;9(2):e21977. doi: 10.2196/21977 (PMC7892284; doi:10.2196/21977)
Supplement: Multimedia Appendix 6 [file mhealth_v9i2e21977_app6.pdf]

Questionnaire: expectations and experiences with the COPD app

Expectations (7-point scale, 1: totally disagree to 7: totally agree)

1. By using the app, I will have more control over my condition COPD
2. By using the app, I will better recognize complaints and symptoms of my condition COPD
3. By using the app, I will know better what to do when my complaints and symptoms get worse
4. It will take no effort to use the COPD app
5. People in my direct environment (eg, family and friends) will stimulate me to use the COPD app
6. I have enough skills (with the tablet or smartphone) to use the COPD app
7. I will get enough help using the COPD app
8. I intend to use the COPD app

Experiences (7-point scale, 1: totally disagree to 7: totally agree)

9. By using the app, I have more control over my condition COPD
10. By using the app, I recognize complains and symptoms of my condition COPD better
11. By using the app, I know better what to do when my complaints and symptoms get worse
12. It takes no effort to use the COPD app
13. People in my direct environment (eg, family and friends) stimulated me to use the COPD app
14. I have enough skills (with a smartphone or tablet) to use the COPD app
15. I get enough help using the COPD app
16. I intend to keep using the COPD app
