# Supplementary material for: Effectiveness of a Mobile Health and Self-Management App for High-Risk Patients With Chronic Obstructive Pulmonary Disease in Daily Clinical Practice: Mixed Methods Evaluation Study
Source: JMIR Mhealth Uhealth. 2021 Feb 4;9(2):e21977. doi: 10.2196/21977 (PMC7892284; doi:10.2196/21977)
Supplement: Multimedia Appendix 7 [file mhealth_v9i2e21977_app7.pdf]

Table 4. Use of the COPD app (N=39)

Use	Week 1	2	3	4	5	6	7	8	9-20[a]
**COPD app**									
Pageclicks, Median (IQR)	6.0 (3.5 – 10.0)	3.0 (2.0 – 5.0)	2.0 (2.0 – 5.0)	2.0 (1.0 – 3.5)	2.0 (1.0 – 4.5)	2.0 (1.0 – 3.0)	2.0 (1.0 – 3.0)	2.0 (1.0 – 3.5)	3.0 (1.0 – 6.0)
App opened, n (%)	39 (100)	35 (90)	33 (84)	32 (82)	31 (79)	31 (79)	31 (79)	31 (79)	30 (79)
**Information**									
**About the app**									
Pageclicks, Median (IQR)	2.0 (0.0 – 5.0)	0.0 (0.0 – 0.0)	0.0 (0.0 – 0.0)	0.0 (0.0 – 0.0)	0.0 (0.0 – 0.0)	0.0 (0.0 – 0.0)	0.0 (0.0 – 0.0)	0.0 (0.0 – 0.0)	0.0 (0.0 – 0.0)
Information opened, n (%)	27 (69)	7 (18)	3 (8)	1 (3)	2 (5)	1 (3)	0 (0)	0 (0)	6 (16)
**Subpage: about the app**									
Pageclicks, Median (IQR)	2.0 (0.0 – 4.0)	0.0 (0.0 – 0.0)	0.0 (0.0 – 0.0)	0.0 (0.0 – 0.0)	0.0 (0.0 – 0.0)	0.0 (0.0 – 0.0)	0.0 (0.0 – 0.0)	0.0 (0.0 – 0.0)	0.0 (0.0 – 0.0)
Information opened, n (%)	22 (56)	5 (13)	3 (8)	0 (0)	1 (3)	1 (3)	0 (0)	0 (0)	6 (16)
**About COPD**									
Pageclicks, Median (IQR)	1.0 (0.0 – 3.0)	0.0 (0.0 – 0.0)	0.0 (0.0 – 0.0)	0.0 (0.0 – 0.0)	0.0 (0.0 – 0.0)	0.0 (0.0 – 0.0)	0.0 (0.0 – 0.0)	0.0 (0.0 – 0.0)	0.0 (0.0 – 5.0)
Information opened, n (%)	22 (56)	7 (18)	8 (21)	2 (5)	4 (10.3)	4 (10)	1 (3)	1 (3)	15 (39)
**Subpage: about COPD**									
Pageclicks, Median (IQR)	0 (0.0 – 2.0)	0.0 (0.0 – 0.0)	0.0 (0.0 – 0.0)	0.0 (0.0 – 0.0)	0.0 (0.0 – 0.0)	0.0 (0.0 – 0.0)	0.0 (0.0 – 0.0)	0.0 (0.0 – 0.0)	0.0 (0.0 – 4.0)
Information opened, n (%)	16 (41)	7 (18)	7 (18)	1 (3)	3 (8)	4 (10)	1 (3)	1 (3)	15 (39)

**Nutrition**									
Pageclicks, Median (IQR)	1.0 (0.0 – 6.0)	0 (0.0 – 1.0)	0 (0.0 – 2.0)	0.0 (0.0 – 0.0)	0.0 (0.0 – 0.0)	0.0 (0.0 – 0.0)	0.0 (0.0 – 0.0)	0.0 (0.0 – 0.0)	0.0 (0.0 – 1.0)
Information opened, n (%)	22 (56)	12 (31)	12 (31)	9 (23)	3 (8)	6 (15)	3 (8)	1 (3)	12 (32)
**Subpage: nutrition**									
Median (IQR)	0.0 (0.0 – 3.0)	0.0 (0.0 – 0.0)	0.0 (0.0 – 0.0)	0.0 (0.0 – 0.0)	0.0 (0.0 – 0.0)	0.0 (0.0 – 0.0)	0.0 (0.0 – 0.0)	0.0 (0.0 – 0.0)	0.0 (0.0 – 0.0)
Information opened, n (%)	17 (44)	9 (23)	9 (23)	8 (21)	3 (8)	5 (13)	3 (8)	1 (3)	9 (24)
**Physical activity**									
Pageclicks, Median (IQR)	1.0 (0.0 – 3.0)	0.0 (0.0 – 0.0)	0.0 (0.0 – 1.0)	0.0 (0.0 – 0.0)	0.0 (0.0 – 0.0)	0.0 (0.0 – 0.0)	0.0 (0.0 – 0.0)	0.0 (0.0 – 0.0)	0.0 (0.0 – 0.0)
Information opened, n (%)	24 (62)	30 (77)	11 (28)	6 (15)	2 (5)	4 (10)	1 (3)	4 (10)	8 (21)
**Subpage: physical activity**									
Pageclicks, Median (IQR)	0.0 (0.0 – 1.0)	0.0 (0.0 – 0.0)	0.0 (0.0 – 0.0)	0.0 (0.0 – 0.0)	0.0 (0.0 – 0.0)	0.0 (0.0 – 0.0)	0.0 (0.0 – 0.0)	0.0 (0.0 – 0.0)	0.0 (0.0 – 0.0)
Information opened, n (%)	11 (28)	9 (23)	7 (18)	3 (8)	2 (5)	2 (5)	1 (3)	3 (8)	5 (13)
**Smoking cessation (pageclicks)**									
Pageclicks, Median (IQR)	0.0 (0.0 – 0.0)	0.0 (0.0 – 0.0)	0.0 (0.0 – 0.0)	0.0 (0.0 – 0.0)	0.0 (0.0 – 0.0)	0.0 (0.0 – 0.0)	0.0 (0.0 – 0.0)	0.0 (0.0 – 0.0)	0.0 (0.0 – 0.0)
Information opened, n (%)	7 (18)	5 (13)	0 (0)	1 (3)	2 (5)	3 (8)	1 (3)	1 (3)	4 (11)
**Subpage: smoking cessation**									
Pageclicks, Median (IQR)	0.0 (0.0 – 0.0)	0.0 (0.0 – 0.0)	0.0 (0.0 – 0.0)	0.0 (0.0 – 0.0)	0.0 (0.0 – 0.0)	0.0 (0.0 – 0.0)	0.0 (0.0 – 0.0)	0.0 (0.0 – 0.0)	0.0 (0.0 – 0.0)

Information opened, n (%)	7 (18)	3 (8)	0 (0)	1 (3)	1 (3)	1 (3)	1 (3)	1 (3)	1 (3)
**Lung Attack Action Plan**									
Pageclicks, Median (IQR)	1.0 (0.0 – 1.0)	0.0 (0.0 – 0.0)	0.0 (0.0 – 0.0)	0.0 (0.0 – 0.0)	0.0 (0.0 – 0.0)	0.0 (0.0 – 0.0)	0.0 (0.0 – 0.0)	0.0 (0.0 – 0.0)	0.0 (0.0 – 0.0)
Information opened, n (%)	22 (56)	3 (8)	2 (5)	0 (0)	0 (0)	2 (5)	0 (0)	1 (3)	6 (15)
**Video consultation**									
Pageclicks, Median (IQR)	0.0 (0.0 – 1.0)	0.0 (0.0 – 0.0)	0.0 (0.0 – 0.0)	0.0 (0.0 – 0.0)	0.0 (0.0 – 0.0)	0.0 (0.0 – 0.0)	0.0 (0.0 – 0.0)	0.0 (0.0 – 0.0)	0.0 (0.0 – 0.0)
Information opened, n (%)	12 (31)	2 (6)	3 (8)	1 (3)	3 (8)	1 (3)	0 (0)	1 (3)	3 (8)

[a] Mean week 9 – 20, n=38
