# Supplementary material for: Effectiveness of a Mobile Health and Self-Management App for High-Risk Patients With Chronic Obstructive Pulmonary Disease in Daily Clinical Practice: Mixed Methods Evaluation Study
Source: JMIR Mhealth Uhealth. 2021 Feb 4;9(2):e21977. doi: 10.2196/21977 (PMC7892284; doi:10.2196/21977)
Supplement: Multimedia Appendix 8 [file mhealth_v9i2e21977_app8.pdf]

Table 5. Patient Satisfaction (N=38)

Satisfaction statements	Week 8, n (%)[a]
**User-friendliness**	
Log in to the COPD app is easy	27 (93)
The COPD app is:	
…easy to use	26 (93)
…well-structured	26 (93)
**Lung Attack Action Plan**	
…is easy to find	27 (96)
…is easy to use	25 (93)
…helped me	18 (67)
**Information**	
The information in the COPD app is understandable	27 (93)
I prefer receiving my information via video instead of text	16 (57)
I am satisfied with the information I received about:	
…the condition COPD	23 (82)
…my daily and extra medication	24 (86)
…breathing techniques	25 (89)
…nutrition	29 (100)
…physical activity	26 (93)
… the advantages of smoking cessation	19 (95)
There is too much information available in the COPD app	9 (33)
I prefer to receive more frequent reminders in the app, regarding new information or questionnaires	16 (57)
**Video consultation**	
I am satisfied with video consultation	18 (78)
I could hear and see the nurse clearly during video consultation	16 (70)
I had problems using video consultation	11 (38) [b]
By using video consultation, I saved time because I did not have to come to the hospital	19 (66) [b]

[a] Valid percentage of patients that (totally) agree (≥5 on 7-point scale)..

[b] yes/no question
