# Supplementary material for: Effectiveness of a Mobile Health and Self-Management App for High-Risk Patients With Chronic Obstructive Pulmonary Disease in Daily Clinical Practice: Mixed Methods Evaluation Study
Source: JMIR Mhealth Uhealth. 2021 Feb 4;9(2):e21977. doi: 10.2196/21977 (PMC7892284; doi:10.2196/21977)
Supplement: Multimedia Appendix 9 [file mhealth_v9i2e21977_app9.pdf]

Table 6. Self-management (N=38)

| **PIH domains** | **Baseline**<br><br>EMM[a]<br>(95% CI) | **8 weeks**<br><br>EMM[a]<br>(95% CI) | **20 weeks**<br><br>EMM[a]<br>(95% CI) | **Change over time**<br>*P*-value[b] |
|---|---|---|---|---|
| Knowledge and coping | 5.2 (4.8 – 5.6) | 5.6 (5.3 – 6.0) | 5.9 (5.5 – 6.3) | *P*=.04 |
| Recognition and management of symptoms, adherence to treatment | 7.0 (6.6 – 7.3) | 7.2 (6.9 – 7.5) | 7.4 (7.1 – 7.6) | *P*=.14 |

[a] Estimated Marginal Means (EMM), Confidence Interval (CI)

[b] Lineair Mixed Model
