# Supplementary material for: Effectiveness of a Mobile Health and Self-Management App for High-Risk Patients With Chronic Obstructive Pulmonary Disease in Daily Clinical Practice: Mixed Methods Evaluation Study
Source: JMIR Mhealth Uhealth. 2021 Feb 4;9(2):e21977. doi: 10.2196/21977 (PMC7892284; doi:10.2196/21977)
Supplement: Multimedia Appendix 10 [file mhealth_v9i2e21977_app10.pdf]

Table 7. Expectations and Experiences with the COPD App

Expectations and Experiences	Baseline (N=39), n (%)[a]	8 weeks (N=38), n (%)[a]	20 weeks (N=37), n (%)[a]
More control over my treatment	27 (73)	18 (56)	20 (67)
Better able to recognize symptoms and complaints	31 (84)	23 (72)	21 (70)
Know what to do when my complaints get worse	31 (84)	23 (72)	23 (77)
It takes no effort to use the COPD app	27 (73)	26 (84)	26 (90)
People in my direct environment stimulate me to use the COPD app	29 (78)	14 (45)	12 (40)
I have enough skills to use the COPD app	25 (68)	26 (87)	25 (83)
I will get enough help using the COPD app	28 (76)	17 (57)	13 (45)
I intend to use/keep using the COPD app	34 (94)	19 (63)	20 (69)

[a] Valid percentage of patients that (totally) agree (≥5 on 7-point scale, 1: totally disagree to 7: totally agree)
